# Treatment outcome of intravenous artesunate in patients with severe malaria in the Netherlands and Belgium

**DOI:** 10.1186/1475-2875-11-102

**Published:** 2012-03-31

**Authors:** Annemarie R Kreeftmeijer-Vegter, Perry J van Genderen, Leo G Visser, Wouter FW Bierman, Jan Clerinx, Cees KW van Veldhuizen, Peter J de Vries

**Affiliations:** 1 Department of Internal Medicine, Division of Infectious Diseases, Academic Medical Center, Meibergdreef 9, 1105AZ Amsterdam, the Netherlands; 2 ACE Pharmaceuticals B.V., Schepenveld 41, 3891 ZK Zeewolde, the Netherlands; 3 Department of Internal Medicine, Harbour Hospital and Institute for Tropical Diseases, Haringvliet 72, 3011 TG Rotterdam, the Netherlands; 4 Department of Infectious Disease, Section Travel Medicine, Leiden University Medical Centre, Albinusdreef 2, 2333 ZA Leiden, the Netherlands; 5 Department of Internal Medicine, University Medical Centre Groningen, Hanzeplein 1, 9713 GZ Groningen, the Netherlands; 6 Department of Clinical Sciences, Institute of Tropical Medicine Antwerp, Nationalestraat 155 B, 2000 Antwerp, Belgium

**Keywords:** Intravenous artesunate, Severe malaria, Parasite clearance, Named patient program, European traveller

## Abstract

**Background:**

Intravenous (IV) artesunate is the treatment of choice for severe malaria. In Europe, however, no GMP-manufactured product is available and treatment data in European travellers are scarce. Fortunately, artesunate became available in the Netherlands and Belgium through a named patient programme. This is the largest case series of artesunate treated patients with severe malaria in Europe.

**Methods:**

Hospitalized patients treated with IV artesunate between November 2007 and December 2010 in the Netherlands and Belgium were retrospectively evaluated. Patient characteristics, treatment and clinical outcome were recorded on a standardized form and mortality, parasite clearance times and the occurrence of adverse events were evaluated.

**Results:**

Of the 68 treated patients, including 55 with severe malaria, two patients died (2/55 = 3.6%). The mean time to 50% parasite clearance (PCT50), 90% and 99% were 4.4 hours (3.9 - 5.2), 14.8 hours (13.0 - 17.2), and 29.5 hours (25.9 - 34.4) respectively. Artesunate was well tolerated. However, an unusual form of haemolytic anaemia was observed in seven patients. The relationship with artesunate remains uncertain.

**Conclusions:**

Data from the named patient programme demonstrate that IV artesunate is effective and well-tolerated in European travellers lacking immunity. However, increased attention needs to be paid to the possible development of haemolytic anaemia 2-3 weeks after start of treatment.

Treatment of IV artesunate should be limited to the period that IV treatment is required and should be followed by a full oral course of an appropriate anti-malarial drug.

## Background

Intravenous (IV) quinine or a mixture of four cinchona alkaloids (Quinimax)^® ^were, for many years, the only available drugs in Europe for the treatment of imported severe malaria. In the US, where quinine is not available, quinidine was used for this indication. Quinine IV has a narrow therapeutic window with significant adverse effects, such as hypotension, cardiac arrhythmia, acoustic problems with temporary deafness and hypoglycaemia. The arrhythmogenic effects of quinidine are even more pronounced.

In the early 1970s, artemisinin, isolated from the Chinese herb Qinghao (sweetworm or *Artemisia annua*), was found to be a very potent anti-malarial agent. Artemisinin drugs have a broad stage specificity of action against all of the erythrocytic stages of the parasite, induce fast parasite clearance and prevent maturation and sequestration of parasites [1]. For parenteral administration, semi-synthetic derivatives of artemisinin were developed with even higher intrinsic activity than the parent compound. The water-soluble sodium artesunate was developed for IV administration.

The two largest trials ever conducted for severe malaria in endemic regions showed that, in both adults and children, treatment with IV artesunate is superior to IV quinine [2,3]. The mortality rate among quinine treated patients was 22% and 10.9% in the SEAQUAMAT and AQUAMAT studies respectively; for artesunate this was 15% and 8.5%, a significant reduction of 35% and 22.5% respectively. Patients with hyperparasitaemia (> 10% of RBC) had a significantly greater treatment effect with artesunate than non-hyperparasitaemic patients. Artesunate was also better tolerated, safer and easier to use than quinine. The life-saving benefit of artesunate in severe malaria was recognized by the WHO in 2006, and IV artesunate is since then the treatment of choice for severe falciparum malaria [4].

The mortality of severe malaria among European patients is lower than in endemic countries. A recent study by Bruneel and colleagues [5] showed a mortality rate of 10.5% among French patients with severe malaria treated with IV quinine. Artesunate has not yet been studied widely in European travellers with severe malaria, with no or partial immunity, although some case series have been described recently [6,7].

The main barrier for the use of IV artesunate in Europe and the US is the absence of a product that is manufactured under Good Manufacturing Practices (GMP). The Chinese manufacturer (Guilin Pharmaceutical Company Ltd., Shanghai, China) of the product that was also used in the SEAQUAMAT and AQUAMAT studies recently improved the production process, with support of the Medicine for Malaria Venture (MMV). This convinced the WHO to list it as prequalified medicinal product [8]. This ensures that manufacturing of the product has been evaluated and inspected by WHO and complies with WHO requirements for essential drugs. Nevertheless, this is not the same as GMP certification and in both the EU and the US, IV artesunate does not have a market authorization. A salient fact is that IV quinine is also not registered in most industrialized countries, where it is mainly available through extemporaneous preparation by hospital pharmacies, and there is no IV quinine formulation on the WHO list of prequalified medicinal products.

The Guilin IV artesunate product was made available in the Netherlands following a specific article in the Dutch Medicines Act. Import and quality control, based on a monograph compliant to the European Pharmacopoeia and the United States Pharmacopoeia, is performed by a Dutch company (ACE Pharmaceuticals B.V., Zeewolde, the Netherlands), which distributes the product under the trade name Malacef 60^®^. Malacef received an orphan designation (EU/3/07/430) by the European Medicines Agency in February 2007. It is available through a named patient programme. The product, kept in emergency stock in the hospital pharmacy, is prescribed to an individual patient accompanied by a medical statement.

The objective of this study was to collect safety and efficacy data of patients treated with IV artesunate. All Dutch patients treated with IV artesunate since its introduction in 2007 were traced and their data on safety and efficacy were actively pursued. For Belgian patients, this IV artesunate product became available in the beginning of 2009 and data were collected from all traceable patients.

## Methods

### Patients

All Dutch hospitalized patients, treated with IV artesunate between November 2007 and December 2010 and Belgian patients treated between January 2009 and December 2010 and of whom data were available, were traced.

### Data collection

Through ACE Pharmaceuticals, the distributor of IV artesunate, the addresses of the hospital pharmacies who had ordered Malacef 60^® ^were obtained. The pharmacies or infectious diseases specialists in those hospitals were proactively contacted and through these the physicians who had prescribed Malacef 60^®^. They were requested to fill out a standardized and anonymized case report form (CRF) taking the data from the patients' medical charts. The following data were collected: demographic data and travel-related history; clinical and laboratory data at presentation, the dosage of artesunate as well as that of other administered anti-malarials and supportive therapy; complications and outcome data, including malaria parasite counts. In all hospitals parasite counts were performed using Giemsa stained thick and thin blood smears and density was either expressed as a percentage of parasitized erythrocytes on a thin film or as the number of *Plasmodium falciparum trophozoites* per 100 white blood cells on a thick film. The parasite load was calculated from these figures using the actual number of WBCs or RBCs counted in the blood sample.

### Definitions and criteria

This was not a prospective trial and so there were no fixed criteria for indication or patient selection. All criteria were applied retrospectively for the purpose of description and analysis of the data. This study did not require approval from an ethics committee according to the Dutch Medical Research Involving Human Subjects Act (WMO).

The indication to administer IV artesunate, as well as the criteria used to define severe malaria, were not exactly similar in all hospitals. All used the WHO criteria[9]: impaired consciousness, multiple convulsions (> 2 episodes/24 h), respiratory distress or pulmonary oedema, circulatory collapse (systolic blood pressure < 70 mm Hg), haemoglobinuria, abnormal spontaneous bleeding, hypoglycaemia (glucose < 2.2 mmol/l), metabolic acidosis (plasma bicarbonate < 15 mmol/l, pH < 7.25), severe anaemia (haemoglobin (Hb) < 3.1 mmol/l), hyperparasitaemia (> 5%), hyperlactataemia (lactate > 5 mmol/l), renal impairment (serum creatinine > 265 μmol/l), jaundice (serum bilirubin > 50 μmol/L). In most hospitals malaria was considered severe with one or more of these criteria; in one Belgian hospital artesunate was used only for "very severe" malaria, with either parasitaemia > 10% or three or more criteria of severity.

### Data handling and statistical analysis

Since this is an observational study which is not easily comparable to other studies, sample size calculation does not lead to precise estimates of power. Therefore the sample size of this study was mainly based on pragmatic and logistic reasons but also on comparison to data given in literature. For example, given the reduction of mortality in the SEAQUAMAT and AQUAMAT trials and the mortality rate among European patients treated with quinine [5], the mortality expected among European patients with severe malaria, could be in the order of 7%. That would imply inclusion of minimally 50 patients in this study.

All extracted data were entered into a Microsoft Access^® ^database and reviewed for inconsistencies. Routine statistical analyses were carried out using IBM SPSS Statistics 18 (IBM Inc, Chicago, IL). Descriptive statistics were used to summarize baseline values and demographic data. Normality was assessed by the Kolmogorov-Smirnov test. Population kinetic modelling is the method of choice for unbalanced repeated measurements [10]. Parasite clearance time (PCT)50%, PCT90%, and PCT99% were defined as the time (in hours) to obtain a 50%, 90%, and 99% reduction in parasite burden after start of artesunate treatment. They were estimated by linear mixed effects population modelling of the (mono-exponential) log-linear time course of the parasitaemia using maximum likelihood techniques. No additional covariates or factors were included in the model. From this, the population mean values of PCT 50% PCT90% and PCT99% were calculated. The precision of the population estimates is given by their 95% confidence intervals.

## Results

### Patient characteristics

From November 2007 to December 2010, 68 patients with imported malaria, hospitalized for parenteral treatment with artesunate were traced; 52 in the Netherlands and 16 in Belgium. In all but two patients, malaria was acquired in Africa. The majority (57%) of the patients were non-immune travellers who had used no or inadequate malaria chemoprophylaxis. Their clinical presentation is showed in Table 1.

**Table 1:** General characteristics on admission

Characteristics	Value
**Median age (range), y**	

Adult (n = 65)	46.9 (18.4 - 83.6)

Child (n = 3)	4.8 (2.8 - 5.2)

**Men, n (%)**	47 (69)

**Continent of birth, n (%)**	

Europe	34 (50)

Africa	27 (40)

Other	7 (10)

Immunity status*, n (%)	

Non-immune	39 (57)

Partially immune	29 (43)

Semi immune	0 (0)

**Chemoprophylaxis, n (%)**	

Adequate	1 (2)

Inadequate	58 (85)

Absent	49 (72)

Inappropriate	9 (13)

Unknown	9 (13)

**Indication for artesunate, n (%)**	

Severe malaria^§ ^(> 1 of the following criteria)	51 (75)

Impaired consciousness	18 (26)

Multiple convulsions (> 2 episodes/24 h)	0 (0)

Respiratory distress or pulmonary edema	4 (6)

Shock (systolic blood pressure < 70 mm Hg)	6 (9)

Haemoglobinuria	2 (3)

Abnormal bleeding	1 (2)

Hypoglycaemia (glucose < 2.2. mmol/L)	1 (2)

Acidaemia (pH < 7.25)	3 (4)

Acidosis (plasma bicarbonate < 15 mmol/L)	4 (6)

Anaemia (Hb < 3.1 mmol/l or haematocrit < 15%)	0 (0)

Hyperparasitaemia (> 5%)	33 (49)

Hyperlactataemia (lactate > 5 mmol/L)	8 (12)

Renal impairment (creatinine > 265 μmol/L)	7 (11)

Jaundice (bilirubin > 50 μmol/L)	24 (35)

Non severe malaria on admission	17 (25)

Clinical deterioration to severe malaria	4 (6)

Parasitaemia between 2-5%	4 (6)

Unable to take oral medication^†^	5 (7)

Other^‡^	4 (6)

* estimated as described earlier by Van Genderen et al. 2010 [11] Non-immune: European travellers and tourists; partially immune: adult immigrants from a malaria endemic country living in the Benelux; semi-immune: patients who had been living in a malaria-endemic area for at least 2 years preceding the time of diagnosis

^§ ^One patient was diagnosed with a mixed infection of P.*falciparum *and P. *malariae*

^†^One patient was diagnosed with *P.ovale*, one with *P. vivax*

^‡ ^One patient was diagnosed with *P. vivax*

*Plasmodium falciparum *infection was diagnosed in 65 patients, one of these had a mixed infection with *P. malariae*. Two patients were microscopically diagnosed with a *P. vivax* monoinfection, and one with a *P. ovale *infection. 55 patients (81%) were classified with severe malaria (one or more WHO criteria), four of whom had non severe malaria on admission, but deteriorated to severe malaria while receiving anti-malarial treatment other than IV artesunate. The characteristics on admission are summarized in Table 2.

**Table 2:** Clinical and laboratory findings on admission

Characteristic	Number of patients (%)	Value
Mean weight (SD), kg	53 (78)	73.2 (20)

Mean temperature (SD), °C	60 (88)	38.6 (1.3)

Mean respiratory rate (SD), breaths/min	16 (24)	25 (8)

Mean heart rate (SD), beats/min	64 (94)	106 (18)

Mean systolic blood pressure (SD), mm Hg	63 (93)	115 (19)

Mean diastolic blood pressure (SD), mm Hg	63 (93)	66 (13)

Median parasitaemia (range),% parasitized erythrocytes	68 (100)	5.0 (0.05-37.4)

Median haemoglobin (range), mmol/L	68 (100)	8.0 (3-10.2)

Median haematocrit (range),%	66 (97)	38 (18-50)

Mean erythrocytes (SD), × 10^12 ^cells/L	51 (75)	4.2 (1.2)

Median leukocyte count (range), × 10^9^cells/L	68 (100)	5.2 (2.5-13.2)

Median serum urea (range), mmol/L	66 (97)	10 (2-94)

Median serum creatinine (range), μmol/L	68 (100)	92 (3-1081)

Median total bilirubin (range), μmol/L	60 (88)	42 (0-299)

Median sodium (range), mmol/L	68 (100)	134 (121-146)

Median potassium (range), mmol/L	65 (96)	3.8 (2.7-41.0)

Median glucose (range), mmol/L	61 (90)	6.4(2.0-18.4)

Median pH (range)	35 (51)	7.45 (6.71-7.57)

Median base excess (range), mmol/L	40 (59)	0.8 (-18.0-28.6)

Mean HCO_3_^- ^(SD), mmol/L	44 (65)	21.3 (4.9)

Median plasma lactate (range), mmol/L	35 (51)	2.8 (9.0-34.0)

### Treatment

Most patients (84%) received the recommended dose of 2.4 mg/kg bodyweight of artesunate on admission and 12 and 24 hours later and then daily thereafter until they were able to complete the treatment with a full oral course of mainly atovaquone-proguanil (AP, Malarone ^®^) or artemether-lumefantrine (AL, Riamet ^®^), as shown in Table 3.

**Table 3:** Administered treatment

Variable	Number of patients (n_total _= 68)
**Initial treatment, n (%)**	

Artesunate	43 (63)

monotherapy	39 (57)

+ clindamycin	2 (3)

+ doxycycline	2 (3)

Quinine	19 (28)

AP	2 (3)

AL	2 (3)

SP + doxycycline	1 (2)

Unknown oral anti-malarial	1 (2)

**Artesunate**	

Median number of doses (range)	3 (1-7)

Median cumulative dose (range), mg	600 (120-1320)

Median cumulative dose/bodyweight (range), mg/kg	7.5 (2.3-18.1)

**Oral anti-malarial drug after artesunate, n (%)**	

AP	41 (60)

AL	20 (29)

Doxycycline	2 (3)

Doxycycline + Quinine	1 (2)

Clindamycin + Quinine	1 (2)

AP + mefloquine	1 (2)

Primaquine	1 (2)

Mefloquine	1 (2)

**Supportive care, n (%)**	

Erythrocytapheresis	4 (6)

Exchange transfusion	13 (19)

Blood transfusion	9 (13)

Platelets transfusion	1 (2)

Mechanical ventilation	6 (9)

Haemodialysis	5 (7)

AP: atovaquone-proguanil; AL: artemether-lumefantrine; SP: sulphadoxine-pyrimethamine;

Before receiving artesunate, 25 patients (37%) received another anti-malarial drug. The median number of IV dosages of artesunate was 3 (range 1-7) doses. The median cumulative artesunate dose was 600 mg (range 120-1320 mg) or 7.5 mg/kg (range 2.3-18.1) mg/kg. The median time between diagnosis of malaria and first dose of artesunate was 4.0 hours (range 0-47 hours). Treatment was changed to oral anti-malarial drugs after a time of 2 days (range 0-6 days) after first dose of artesunate. The majority received either AP (60%) or AL (29%).

### Efficacy

All patients survived the acute malaria episode. Two of 55 patients (3.6%) with severe malaria died. The non severe malaria patients all survived but were excluded from mortality calculation. One patient died of an iatrogenic complication (IV catheter related haemorrhage) 4 days after complete parasite clearance; the other succumbed to a suspected lung embolism 8 days after parasite clearance (Table 4). 42 (62%) patients were admitted to the ICU with a median ICU stay of 2 days (range 1-35 days). Median hospital stay was 4.5 days (range 1-76 days). Follow up was performed in 49 patients (72%), with a median follow up time of 23 days (range 6 - 203 days) after first day of hospitalization. The mean values for PCT50%, PCT90% and PCT99% were 4.4 hours (95% confidence interval 3.9 - 5.2 h), 14.8 hours (95% confidence interval 13.0 - 17.2 h), and 29.5 hours (95% confidence interval 25.9 - 34.4 h) respectively.

**Table 4:** Outcome

Endpoint	Number of patients (n = 68)
Mortality, n (%)	

among severe malaria,% (n = 55)	2* (3.6)

Mean parasite clearance times (95% CI), hours^†^	

PCT_50%_	4.4 (3.9 - 5.2)

PCT_90%_	14.8 (13.0 - 17.2)

PCT_99%_	29.5 (25.9 - 34.4)

ICU admittance, n (%)	42 (62)

Median duration of ICU stay (range), days	2 (1-35)

Median duration of hospital stay (range), days	4.5 (1-76)

PCT50%, PCT90%, and PCT99% are the parasite clearance times, defined as the time (in hours) to obtain a 50%, 90%, and 99% reduction in parasite count after start of artesunate treatment, respectively

* One patient still suffered from a delirious state and in an unobserved moment he inadvertently removed his haemofiltration catheter from the femoral vein. He died due to a loss of blood. A second patient died shortly after sudden collapse during defecation and pulmonary embolism was suspected but not confirmed as autopsy was not permitted. It occurred while the patient was treated with subcutaneous low molecular weight heparin

^† ^Calculated from 57 patients with the following conditions:

■ the limit of detection was set at 10 parasites/μL. Non detectable parasite counts were not included in the log linear effects model

■ when more than one slide was made before artesunate treatment was given, the oldest slides were excluded from analysis. The value nearest to T_0 _was used

■ for repeated parasite counts around the detection limit, only the first value was included in the model

■ Malaria cases caused by other species than *P. falciparum *were excluded for estimation of parasite clearance rates

■ In most patients (88%) parasite counts were performed at least twice in the first 24 hours (range 1-7 times)

### Safety

**Figure 1:**
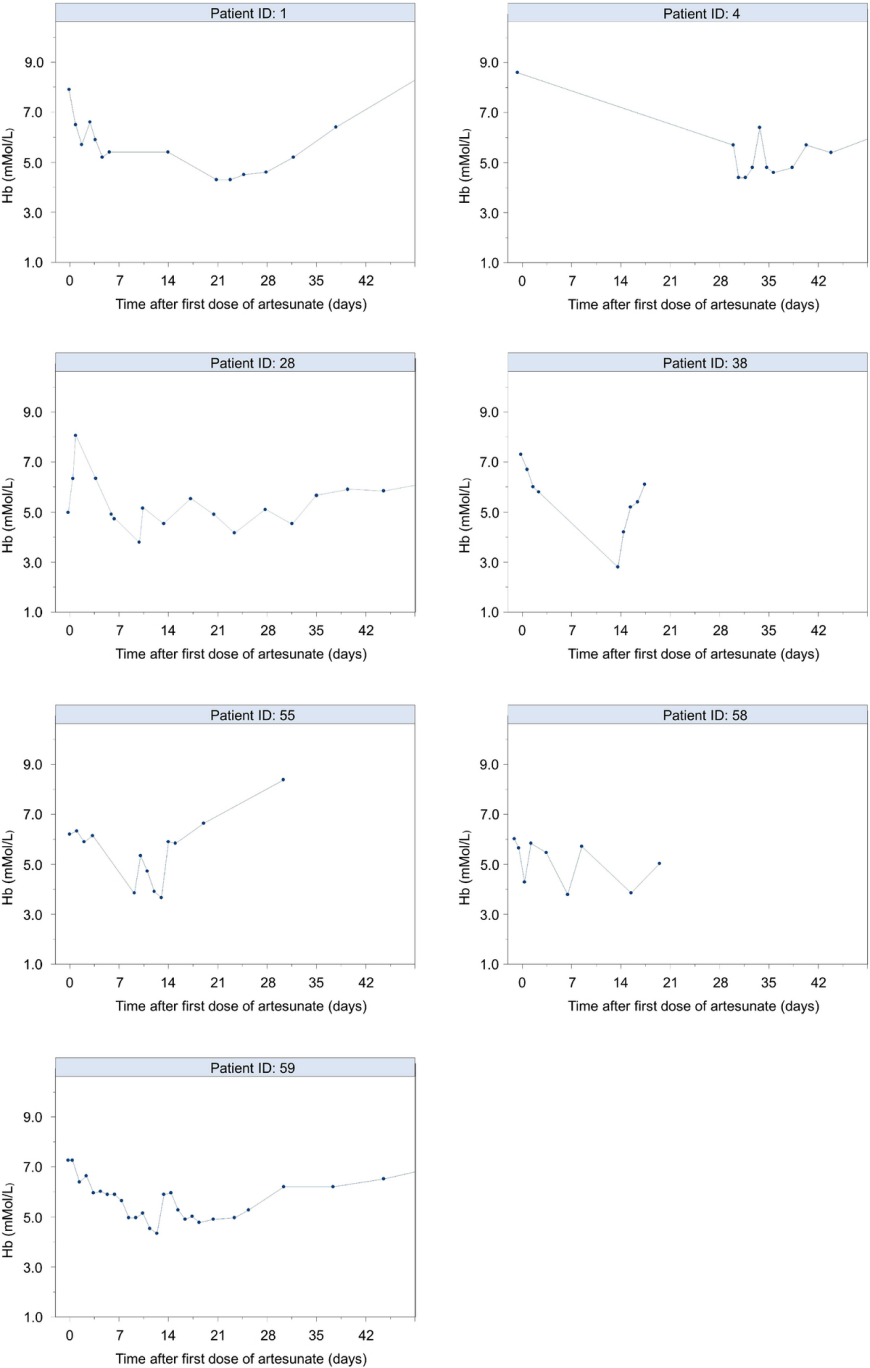
**Haemoglobin values of patients with haemolysis after being treated for severe malaria with IV artesunate**.

All reported complications are listed in Table 5. Most recorded complications were compatible with the clinical findings in severe malaria and already present before initiation of IV artesunate. These complications were not recorded as drug related. Noteworthy was the occurrence of late onset haemolytic anaemia in six patients with severe malaria (Table 6), characterized by increased reticulocyte counts, unconjugated bilirubin and lactate dehydrogenase and decreased haptoglobin and haemoglobin (Hb) values. Hb nadir occurred between 7 and 31 days after treatment initiation (Figure 1). In two patients, haemolysis started when fever had not yet subsided, in the other four there was complete parasite and fever clearance. In five patients, the pattern of haemolysis was characterized by a decrease of the Hb value or a failure of the Hb value to recover during week 2. The remaining patient (nr. 4) developed an unexplained neurological syndrome accompanied by signs of haemolysis with the Hb nadir 30 days after starting artesunate treatment. Also, one patient (nr. 28) experienced persistent haemolysis until 7 weeks after artesunate treatment which required a total of 7 blood transfusions (Table 6).

**Table 5:** Complications

Complication	Number of patients (%)	Recovery*
***Haematological, n (%) ***		

Abnormal bleeding	1 (2)	Recovered

Anemia	32 (47)	
hemolytic^†^	7 (10)	Recovered in all
severe (Hb< 3.6 mmol/l)	2 (3)	Recovered in all
moderately severe (3.7 - 6.1 mmol/l)	23 (34)	Recovered in 11/11 ^‡^

Thrombocytopenia (< 20 giga/L)	16 (24)	Recovered in all

Hemoglobinuria	1 (2)	Recovered

***Hepatobiliary, n (%)***		

Elevated liver enzymes (LDH > 2x URL)	31 (46)	Recovery in 14/14^‡^

***Renal, n (%)***		

Acute renal insufficiency	7 (10)	Recovered in 5/5 ^‡^

Acute tubular necrosis	1 (2)	Recovered

Anuria	1 (2)	Not resolved, still in need of dialysis

Elevated creatinine levels (> 20% above URL)	9 (13)	Recovered in 4/4^‡^

***Nervous system, n (%)***		

Anal sphincter dysfunction	1 (2)	Recovered

Aphasia	1 (2)	Recovered

Cerebral edema	1 (2)	Recovered

Coma	2 (3)	Recovered

Convulsion	3 (4)	Recovered

Delirium	2 (3)	Recovered in 1/2 ^§^

Diplopia	1 (2)	Restoring but lost to follow up

Neurological deficit	1 (2)	Recovered

Truncal ataxia	1 (2)	Restoring but lost to follow up

***Cardiovascular, n (%***		

Arterial hypertension	1 (2)	Recovered

Atrial flutter	2 (3)	Recovered

AV Block (1^st ^degree)	1 (2)	Recovered

Prolonged QTc interval	1 (2)	Recovered

Sinus bradycardia	1 (2)	Recovered^║^

Supraventricular tachycardia	1 (2)	Recovered

Thrombophlebitis	2 (3)	Recovered

***Respiratory, n (%)***		

Exercise induced dyspnea	1 (2)	Recovered

Pneumonia	4 (6)	Recovered

Respiratory insufficiency	4 (6)	Recovered

***Other, n (%)***		

Deafness	3 (4)	Recovered in 2/3^¶^

Diarrhoea	3 (4)	Recovered

Fever	1 (2)	Recovered

Furunculosis	1 (2)	Recovered

Hypoglycaemia	1 (2)	Recovered

Peri-anal abcess	1 (2)	Recovered

Rash	2 (3)	Recovered

Reactive arthritis	1 (2)	Recovered

Sepsis	1 (2)	Recovered

Tropical ulcers	1 (2)	Recovered

URL: Upper reference limit reported by laboratory of admitting hospital

* Number of patients that completely recovered/number of patients of whom data was complete (adequate follow up)

^† ^characterized by increased reticulocyte counts, unconjugated bilirubin and lactate dehydrogenase and decreased haptoglobin and haemoglobin (Hb) values. See Table 6 for further details

‡ complication restoring in the remaining patients but patients were not followed until complete recovery (incomplete data)

^§ ^the other patient died

^║ ^patient received pacemaker

^¶^right ear remains deaf in one patient

**Table 6:** Haemolysis in 7 hospitalized malaria patients receiving IV artesunate treatment

Patient (gender, age)	Severe malaria criteria	Anti-malarial treatment	PCT (days)*	Hb nadir(days)*	Additional diagnostics	Treatment for haemolysis
**Late onset hemolysis**						

1 (♂, 53y)	Hyperparasitaemia (34%), impaired consciousness, jaundice	Q AS (2 doses) AP AET	4	4.3 (D20)	Coombs: C3d+	None

4 (♀, 50y)	Hyperparasitaemia (19%), impaired consciousness, jaundice, acidosis, hyperlactaemia, renal impairment	AS (4 doses) AP MET	3	4.4 (D30)	Multiple in the context of an unexplained neurological disorder^‡^; coombs not performed	None

38 (♀, 50y)	Hyperparasitaemia(11%), jaundice	AS (4 doses) AP	3	2.8 (D13)	Coombs: neg; G6PD deficient (heterozygous); Shigellaflexneri dysentery	Transfusion(4 PC)

55 (♀, 44y)	Hyperparasitaemia (37%)	Q AS (3 doses) AL	4	3.8 (D15)	Coombs: IgG +, C3d+	Transfusion(2 × 3 PC) Steroids

58 (♂, 5y)	Hyperparasitaemia (12%), impaired consciousness, shock	Q AS (doses) AL	4(FCT 10 d)	3.8 (D8)	Coombs: neg; hemoculture -	None

59 (♀, 50y)	Hyperparasitaemia (30%), hemoglobinuria, jaundice	AS (doses) AL	10 (FCT 17 d)	4.3 (D13)	Coombs: IgG+, IgM+, hemoculture - G6PD: normal	Transfusion (2 PC) Steroids

**Persistent hemolysis**						

28 (♂, 71y)	Hyperparasitaemia (20%), impaired consciousness, respiratory distress, acidosis, hypoglycaemia, hyperlactaemia, renal impairment	Q AS (doses) AL AET	7^†^	3.7 (D13)	Coombs: neg	Transfusion (7 times, total of 24 PC)

Q: quinine, AS: artesunate, AP: atovaquone-proguanil AL: artemether-lumefantrine, AET: automated exchange transfusion, MET: manual exchange transfusion, PCT: parasite clearance time; FCT: fever clearance time; neg: negative; G6PD: glucose-6-phosphate dehydrogenase; HC: hemoculture; PC: packed red cells; CS: corticosteroids

* number of days after 1^st ^artesunate gift

^† ^Parasitological examination was not performed between day 3 and 6 after first artesunate gift. On day 7, malaria slides were negative

^‡ ^Hospital stay was complicated with disorientation for which patient was treated with haloperidol. Patient left the hospital against medical advice on Day 5 while not recovered fully. Within 4 weeks she was readmitted with an overt status epilepticus and fever. Diazepam, propofol and phenytoin were given after which the epileptic activity ceased. She was intubated and mechanically ventilated for 3 days. An extensive search for infectious (malaria, Epstein-Barrvirus, cytomegalovirus, Herpes simplex virus, Varicella zoster virus, enterovirus, parechovirus) and metabolic causes remained negative. Cerebrospinal fluid culture was negative. MRI of the brain showed diffuse cortical edema and no other abnormalities. She was treated with methylprednisolone after which she started to recover

For the remaining patients with severe malaria there were no reports of haemolysis although Hb values in week 2 were only available for 20 of them. Thus, in theory, the worst case scenario for the frequency of the late onset haemolysis could be in the order of 6/(20 + 6) = 23% (95% confidence interval 7 to 39%). However, we feel that, given the conditions in Belgium and the Netherlands, it is unlikely that severe haemolysis would go unnoticed. There was no difference in median cumulative dose between patients with and without haemolysis (7.2 vs 7.5 mg/kg resp; p = 0.91).

## Discussion

In this study, the Dutch and Belgian experience with the use of IV artesunate on a named patient basis is presented, the largest case series of patients with severe malaria in Europe. Mortality was low and parasite clearance was rapid. Late onset haemolysis was observed, mostly short lived, which confirms recent findings in travellers with severe malaria treated with IV artesunate [7].

In this study, probably all patients treated with artesunate in the Netherlands were captured. The Netherlands is a small country, the professional groups are well organized and there is little chance that patients would have been treated outside our scope. For Belgium, this was different, only patients from two Belgian hospitals were traceable. There is no indication of a selection bias towards the use of artesunate or quinine. Once artesunate was available in hospitals, this became the drug of choice.

A named patient programme cannot substitute for prospective clinical research. Nevertheless, this type of pharmacovigilance studies may capture more information than a prospective clinical trial, especially rare and late onset events. All treating hospitals are large medical centres with high quality care, accredited laboratories and close follow up procedures, ensuring a high reliability and quality of the data.

A limitation of this retrospective study is the heterogeneity of patient characteristics and therapeutic interventions: Twenty-five patients received quinine or other anti-malarial agents before artesunate, while 17 received exchange transfusion or erythrocytapheresis. The decision to treat with IV artesunate is only part of the total routine case management. In Belgium, criteria for administrating IV artesunate are restricted to patients with 'very severe malaria' and vomiting patients intolerant of IV quinine. In the Dutch malaria treatment guidelines ('StichtingWerkgroepAntibioticabeleid' (SWAB), the Dutch Working Party on Antibiotics Policy; http://www.swab.nl), artesunate is treatment of choice in severe malaria, however, when not available, quinine should be administered instead or until artesunate is available. The availability of artesunate increased rapidly in the last few years (seven out of 90 Dutch hospitals had artesunate in stock in 2008, 44 in 2010 (distribution records)), while pre-treatment with quinine has decreased in the same period.

Although the contribution of exchange transfusion has been disputed, it accelerates parasite clearance during quinine treatment [11]. Its benefit during artesunate treatment needs further study. Nonetheless, Dutch SWAB guidelines consider exchange transfusion in patients with > 10% parasitaemia or severe illness with organ failure. In Belgium, erythrocytapheresis was the standard procedure in patients with very high parasitaemia (> 15%) until IV artesunate became available. Since then, it has been largely abandoned.

The cure and survival rates among patients with severe malaria in the present study were high. The death of the two patients was not directly related to malaria and/or the treatment with artesunate. Although sample size of the present study is small and although it is inappropriate to infer the evidence from endemic regions [2,3] and the treatment outcome of quinine in French malaria patients to the present population, it confirms what has been stated before; IV artesunate is an excellent drug for severe malaria, also under the resourceful conditions of case management in the Netherlands and Belgium.

It has been discussed whether the evidence from endemic regions can be generalized to the European population. Patients characteristics, clinical manifestations and supportive care may differ greatly between developing and industrialized countries [12]. It has also been debated whether this would justify or even require a trial comparing artesunate and quinine for European patients. Our current view is that severe malaria is such a rare disease in Europe that sufficient sample sizes cannot be obtained. Furthermore, the question remains whether European physicians would be willing to participate in such a trial now that artesunate is recommended as the treatment of choice for severe malaria by WHO [9].

Severe post-treatment haemolysis occurred unexpectedly in seven patients. This was also observed in 24% of patients treated with IV artesunate in another study [7] in which post-treatment haemolysis was associated with higher doses of artesunate and longer treatment periods. This was not observed in the present study. Four patients received AL as a consolidation treatment. Artemether but also lumefantrine or quinine may induce haemolysis ('blackwater fever') [13,14].

Surprisingly, in the SEAQUAMAT and AQUAMAT trials [2,3], over 3000 patients were treated with IV artesunate, even with higher cumulative doses of artesunate than in the present study, and often also with AL, but no haemolysis was reported. These trials were not designed to capture this late onset event.

In acute falciparum malaria, anaemia and prolonged haemolysis may persist for weeks after elimination of parasites, regardless the treatment given [15,16]. Other factors could also have contributed to the haemolysis. For example, one patient was heterozygous for glucose-6-phosphate dehydrogenase (G6PD) deficiency. Three patients received exchange transfusion in conjunction with anti-malarial treatment. Furthermore, both positive (3 times) and negative (3 times) direct Coombs test results were observed. Anti-erythrocyte antibodies can be secondary to malignancies, autoimmune disorders, transfusion reactions and also to drugs [17] and is rather common after malaria [18]. Drug induced haemolysis with a negative direct Coombs test has also been documented [19].

Since physicians were not alerted to this phenomenon, additional investigations were not performed. It is therefore difficult to assess whether exposure to artesunate was the sole cause. Moreover, the causality of artesunate administration is not easily established by using adverse drug reaction scales, mainly because scores on these scales are largely affected by the relationship in time between drug administration and onset of the adverse event [20]. The haemolysis started long after complete clearance of artesunate [21]. There are several effects of artesunate on cellular biology, including suppressive effects on erythropoiesis and angiogenesis [22]. Recently Berdelle and colleagues demonstrated that artesunate has mutagenic potential [23]. Whether these effects extend to a-nuclear erythrocytes is not known. Another explanation of late onset hemolysis could be a reduced survival of 'pitted' infected erythrocytes [24,25]. This would explain the fact that in this study haemolysis was only seen in patients with very high parasitaemia (11-37%). This is refuted by the fact that not all patients with high parasitaemia develop late onset haemolysis. Moreover, a similar type of hemolysis was also observed in a patient with non-hyperparasitaemic non-severe malaria, treated with AP followed by AL (unpublished data). Whatever the mechanism, a weekly follow up for 4 weeks with Hb measurement and, if not improving, also other parameters of haemolysis, should be performed. More importantly, as the majority of parasites is cleared within 24 hours, and in light of the possible role of artesunate in the development of late onset haemolytic anaemia, the authors would like to recommend to limit treatment to the period that IV treatment is deemed necessary. In practice this is no longer than 48 hours. Treatment should be followed by an adequate and full course of oral anti-malarial treatment.

There is still no marketing authorization for an artesunate product on the European market. This is due to the fact that it is challenging to develop a suitable formulation that meets the requirements of GMP. Several companies are currently working on GMP-conform IV artesunate formulations, while others work on semi-synthetic production of artemisinins [26,27]. Importing drugs into Europe is subject to strict regulations, which ensure that patients would never receive a non-properly tested and released product. There are several companies involved in importing artesunate into Europe.

### Conclusions and recommendation

The excellent efficacy of IV artesunate for severe imported malaria in industrialised countries supports the efforts to make this drug available throughout Europe. The roll-out of IV artesunate should be closely monitored by a pharmacovigilance programme, such as was set up for this study. Meanwhile, increased safeguards with respect to haematological abnormalities should be instituted in the follow-up period after anti-malarial treatment at least once weekly until 4 weeks after initiation of therapy. The fast clearance of parasites warrants the reduction of the length of treatment with artesunate. The authors would, therefore, recommend that treatment with artesunate should be limited to the period that IV treatment is required and should be followed by a full oral course of an appropriate anti-malarial drug.

## Abbreviations

(IV): Intravenous; (PCT): Parasite clearance times; (GMP): Good Manufacturing Practices; (MMV): Medicine for Malaria Venture; (CRF): Case report form; (WMO): Dutch Medical Research Involving Human Subjects Act; (Hb): Haemoglobin; (AP): Atovaquone-proguanil; (AL): Artemether-lumefantrine; (G6PD): Glucose-6-phosphate dehydrogenase

## Competing interests

ARKV is PhD fellow, employed by ACE Pharmaceuticals; CKWVV is pharmacist for ACE Pharmaceuticals B.V.

## Authors' contributions

ARKV, CKWVV and PJDV conceived and designed the study. ARKV and PJDV analysed and interpreted the data. PVG, LV, WB and JC helped acquire the data. All authors were involved in critical revision and approval of the paper.
